# Methodological standards for the development and evaluation of clinical prediction rules: a review of the literature

**DOI:** 10.1186/s41512-019-0060-y

**Published:** 2019-08-22

**Authors:** Laura E. Cowley, Daniel M. Farewell, Sabine Maguire, Alison M. Kemp

**Affiliations:** Division of Population Medicine, School of Medicine, Neuadd Meirionnydd, Heath Park, Cardiff University, Wales, CF14 4YS UK

**Keywords:** Clinical prediction rule, Prediction model, Risk model, Model development, Model validation, Impact studies, Model reporting, Implementation, Diagnosis, Prognosis, Study design

## Abstract

Clinical prediction rules (CPRs) that predict the absolute risk of a clinical condition or future outcome for individual patients are abundant in the medical literature; however, systematic reviews have demonstrated shortcomings in the methodological quality and reporting of prediction studies. To maximise the potential and clinical usefulness of CPRs, they must be rigorously developed and validated, and their impact on clinical practice and patient outcomes must be evaluated. This review aims to present a comprehensive overview of the stages involved in the development, validation and evaluation of CPRs, and to describe in detail the methodological standards required at each stage, illustrated with examples where appropriate. Important features of the study design, statistical analysis, modelling strategy, data collection, performance assessment, CPR presentation and reporting are discussed, in addition to other, often overlooked aspects such as the acceptability, cost-effectiveness and longer-term implementation of CPRs, and their comparison with clinical judgement. Although the development and evaluation of a robust, clinically useful CPR is anything but straightforward, adherence to the plethora of methodological standards, recommendations and frameworks at each stage will assist in the development of a rigorous CPR that has the potential to contribute usefully to clinical practice and decision-making and have a positive impact on patient care.

## Background

The aim of a clinical prediction rule (CPR) is to estimate the probability of a clinical condition or a future outcome by considering a small number of highly valid indicators [1, 2]. CPRs include three or more predictors, from patients’ clinical findings, history or investigation results [3]. Their purpose is to assist clinicians in making decisions under conditions of uncertainty and enhance diagnostic, prognostic or therapeutic accuracy and decision-making, with the ultimate aim of improving the quality of patient care [1, 2, 4]. The predicted probabilities from a CPR allow clinicians to stratify patients into risk groups and help them to decide whether further assessment or treatment is necessary [5]. Some CPRs can help to ‘rule in’ a condition by identifying patients who are very likely to have a condition and who thus require additional diagnostic testing or treatment, whilst others aim to ‘rule out’ a condition by identifying patients who are very unlikely to have a condition, thus reducing unnecessary testing without compromising patient care [2, 4]. CPRs that aim to predict the probability of a condition being present are termed *diagnostic* or *screening* rules; those that aim to predict the probability of a future outcome are termed *prognostic* rules; and those that aim to predict the probability that a specific treatment or intervention will be effective are termed *prescriptive* rules [2].

To maximise the predictive accuracy and clinical utility of CPRs, it is vital that they are rigorously developed, validated and evaluated. However, numerous systematic reviews have demonstrated shortcomings in the methodological quality and reporting of prediction studies, which restricts the CPR’s usefulness in practice [6–15]. Methodological standards for the development of CPRs were originally outlined by Wasson and colleagues [16]. With the increase in popularity of CPRs inspired by the evidence-based medicine movement, these standards have since been modified and updated by a number of authors over the years [3, 4, 17–19]. Experts have provided thorough and accessible overviews of the principles and methods involved in conducting diagnostic and prognostic research [20–32] and devised frameworks to enhance the conduct and interpretation of prediction studies [33–35]. They have also provided guidance and recommendations for researchers to consider when developing and evaluating CPRs, without aiming to dictate how analyses should be conducted. These recognise that there is no clear consensus on many aspects of model development, that the field is continually evolving and that methodological standards will therefore require updating accordingly [36]. Guidelines for the *reporting* of clinical prediction research have also been developed, namely the Transparent Reporting of a multivariable prediction model for Individual Prognosis or Diagnosis (TRIPOD) guidelines [36].

This review aims to outline the stages and methodological standards involved in the development and evaluation of CPRs, illustrated with examples where appropriate.

### Terminology used in this review

In the literature, the term ‘clinical prediction rule’ is used interchangeably with the terms clinical prediction tool [37], clinical decision rule [17], clinical decision tool [38], clinical prediction algorithm [39], prognostic score [40], prognostic model [21], risk prediction model [23], risk model [30], risk score [41], scoring tool [42], scoring system [43] or risk index [44]. Reilly and Evans [32] distinguish between *assistive prediction* rules that simply provide clinicians with diagnostic or prognostic predicted probabilities without recommending a specific clinical course of action, and *directive decision* rules that explicitly suggest additional diagnostic tests or treatment in line with the obtained score. Decision rules intend to directly influence clinician behaviour, while prediction rules intend to help clinicians predict risk without providing recommendations, with the assumption that accurate predictions will lead to better decisions [32]. Some researchers also distinguish between prediction *models* that provide predicted probabilities along the continuum between certified impossibility (*Pi* = 0) and absolute certainty (*Pi* = 1) [45], and prediction *rules* that classify patients into risk groups, by applying a clinically relevant cut-off that balances the likelihood of benefit with the likelihood of harm [19, 46]. Such cut-offs are known as ‘decision thresholds’; a threshold must be applied if a prediction model aims to influence decision-making [19]. In this review, the term ‘clinical prediction rule’ is used to refer to diagnostic, prognostic or prescriptive rules/models derived from multivariable statistical analyses, which predict the probability of a condition or outcome, *with or without* the use of a clinical cut-off or recommendation for further action.

## Stages in the development of clinical prediction rules

**Fig. 1:**
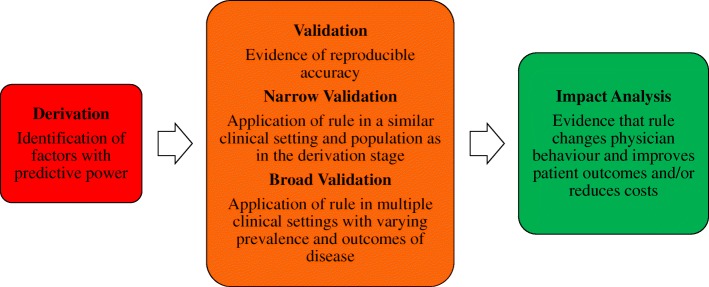
The three main stages in the development and evaluation of clinical prediction rules. Adapted from McGinn, 2016 [47]

It is widely acknowledged in the literature that there are three *main* stages in the development of CPRs (Fig. 1); derivation; external validation; and impact analysis to determine their impact on patient care [4, 20, 22–25, 32, 33]. Stiell and Wells [17] identified a further three important stages, namely identifying the need for a CPR, determining the cost-effectiveness of a CPR and long-term dissemination and implementation of a CPR. Therefore all six stages are summarised in Table 1 and discussed in detail below.

**Table 1:** Stages in the development and evaluation of clinical prediction rules

Stage of development	Methodological standards
Stage 1. Identifying the need for a CPR	• Consider conducting qualitative research with clinicians to determine clinical relevance and credibility of CPR
	• Conduct a systematic review of the literature to identify and evaluate existing CPRs developed for the same purpose
	• Consider updating, validating or testing the impact of existing CPRs
Stage 2. Derivation of a CPR according to methodological standards	Study design for the derivation of a CPR
	• Consider registering the study and publishing a protocol
	• Ensure the dataset is representative of the population for whom the CPR is intended
	• Conduct a prospective multicentre cohort study
	Statistical analysis
	• Conduct multivariable regression analysis (logistic for binary outcomes, Cox for long-term prognostic outcomes) • Identify the model to be used, plus rationale if other methods used
	Missing data
	• Use multiple imputation
	Selection of candidate predictors for inclusion in a multivariable model
	• Only include relevant predictors based on evidence in the literature/clinical experience
	• Aim for a sample size with a minimum of ten events per predictor, preferably more
	• Avoid selection based on univariable significance testing
	• Avoid categorising continuous predictors
	Selection of predictors during multivariable modelling
	• Backward elimination of predictors is preferred
	• Avoid data-driven selection and incorporate subject-matter knowledge into the selection process
	Definition and assessment of predictor and outcome variables
	• Define predictor and outcome variables clearly
	• Consider inter-rater reliability of predictor measurement and potential measurement error
	• Aim for blind assessment of predictor and outcome variables
	Internal validation
	• Use cross-validation or bootstrapping and adjust for optimism
	• Ensure to repeat each step of model development if using bootstrapping
	CPR performance measures
	• Assess and report both calibration and discrimination
	• Consider decision curve analysis to estimate the clinical utility of the CPR
	Presentation of a CPR
	• Report the regression coefficients of the final model, including the intercept or baseline hazard
	• Consider a clinical calculator if the CPR is complex
	Reporting the derivation of a CPR
	• Adhere to the TRIPOD guidelines [36]
Stage 3. External validation and refinement of a CPR	Study design for the external validation of a CPR
	• Conduct a prospective multicentre cohort study
	• Aim for a sample size with a minimum of 100 outcome events, preferably 200
	• Consider using a framework of generalisability to enhance the interpretation of the findings [34]
	Types of external validation
	• Conduct temporal, geographical and domain validation studies to ensure maximum generalisability
	• If multiple validations have been performed, conduct a meta-analysis to summarise the overall performance of the CPR, using a published framework [35]
	Refinement of a CPR: model updating or adjustment
	• Consider updating, adjusting or recalibrating the CPR if poor performance is found in an external validation study
	• Consider further external validation of updated CPRs
	Comparing the performance of CPRs
	• Compare the CPR with other existing CPRs for the same condition
	• Ensure the statistical procedures used for comparison are appropriate; consider a decision-analytic approach
	Reporting the external validation of a CPR
	• Adhere to the TRIPOD guidelines [36]
Stage 4. Impact of a CPR on clinical practice	Study design for an impact analysis
	• Consider whether the CPR is ready for implementation
	• Conduct a cluster randomised trial with centres as clusters, or a before–after study
	• Perform appropriate sample size calculations
	• Consider decision-analytic modelling as an intermediate step prior to a formal impact study
	Measures of impact of a CPR
	• Report the safety and efficacy of the CPR
	• Report the impact of the CPR on clinician behaviour if assessed
	Acceptability of a CPR
	• Evaluate the acceptability of the CPR using the validated OADRI [48], or using qualitative or vignette methods
	Comparison of a CPR with unstructured clinical judgement
	• Compare the sensitivity and specificity of the CPR with clinicians own predictions/decisions
	The four phases of impact analysis for CPRs
	• Follow the framework for the impact analysis of CPRs [33]
	• Ensure extensive preparatory and feasibility work is conducted prior to a formal impact study
	Reporting the impact analysis of a CPR
	• There are currently no published reporting guidelines for impact studies of CPRs; this is an area for future research
Stage 5. Cost-effectiveness	• Conduct a formal economic evaluation, with sensitivity analyses to examine the uncertainty of the model projections
Stage 6. Long-term implementation and dissemination	• Devise and evaluate targeted implementation strategies to ensure maximum uptake
	Barriers and facilitators to the use of CPRs
	• Assess barriers to the use of the CPR and devise strategies to overcome these

*CPR* clinical prediction rule, *TRIPOD* Transparent Reporting of a multivariable prediction model for Individual Prognosis or Diagnosis, *OADRI* Ottawa Acceptability of Decision Rules Instrument

Detailed methodological and practical recommendations pertaining to the three main stages of development have been published, as each requires a different methodological approach [3, 4, 16–36]. These three stages also correspond to increasing hierarchies of evidence, as outlined in Table 2 [4, 32, 33]. A CPR that has been *derived*, but not externally validated, corresponds to the lowest level of evidence and is not recommended for use in clinical practice, except arguably in rare instances when a CPR is developed for use in only one setting. It has been suggested that a CPR that has been successfully externally *validated* in a setting, or population, similar to the one from which it was derived (‘narrow’ validation), can be used cautiously in similar future patients [32]. Similarly, it is proposed that a CPR should be consistently successfully externally validated in multiple settings or populations (‘broad’ validation), before clinicians can use its predictions confidently in future patients [32]. Finally, it is recommended that an *impact analysis* is conducted and that the CPR demonstrates improvements to patient care, before it can be used as a decision rule for the management and treatment of patients [32]. Ideally, the impact of a CPR should also be tested in multiple settings. Impact analysis studies correspond to the highest level of evidence [32].

**Table 2:** Hierarchies of evidence in the development and evaluation of clinical prediction rules

Level of evidence	Definitions and standards of evaluation	Implications for clinicians
Level 1: Derivation of CPR	Identification of predictors using multivariable model; blinded assessment of outcomes.	Needs validation and further evaluation before it is used clinically in actual patient care.
Level 2: Narrow validation of CPR	Validation of CPR when tested prospectively in one setting; blinded assessment of outcomes.	Needs validation in varied settings; may use CPR cautiously in patients similar to derivation sample.
Level 3: Broad validation of CPR	Validation of CPR in varied settings with wide spectrum of patients and clinicians.	Needs impact analysis; may use CPR predictions with confidence in their accuracy.
Level 4: Narrow impact analysis of CPR used for decision-making	Prospective demonstration in one setting that use of CPR improves clinicians’ decisions (quality or cost-effectiveness of patient care).	May use cautiously to inform decisions in settings similar to that studied.
Level 5: Broad impact analysis of CPR used for decision-making	Prospective demonstration in varied settings that use of CPR improves clinicians’ decisions for wide spectrum of patients.	May use in varied settings with confidence that its use will benefit patient care quality or effectiveness.

Adapted from Reilly and Evans 2016 [32]. *CPR* clinical prediction rule

### Stage 1: identifying the need for a clinical prediction rule

Before developing a CPR, researchers need to ensure that there is a clinical need for the rule. CPRs are most valuable when decision-making is challenging, when there is evidence that clinicians are failing to accurately diagnose a condition, and when there are serious consequences associated with an incorrect diagnosis [2, 4]. CPRs are also valuable when there is a need to simplify or speed up the diagnostic or triage process, for example in patients presenting to the emergency department with chest pain and suspected acute cardiac ischaemia [49]. CPRs are most likely to be adopted into clinical practice, and to demonstrate improvements in patient care and reductions in health care costs, when they improve the overall efficiency of clinical practice [17]. For example, ankle injuries are frequently seen in the emergency department. Prior to the implementation of the Ottawa Ankle Rule, clinicians ordered a high proportion of radiographs that were negative for fracture, when the majority of them believed that a fracture was highly unlikely [50]. The rule was found to lead to a reduction in both radiography [51] and health care costs [52], and in one survey 70% of Canadian and UK emergency department clinicians reported frequent use of the rule [53].

Before developing a CPR, researchers should consider whether a new CPR is needed, as many are developed for the same target population or to predict the same outcome [8, 10, 11, 54–57]. The characteristics, performance and level of evidence of existing CPRs should be systematically reviewed using validated search filters for locating prediction studies, and the Critical Appraisal and Data Extraction for Systematic Reviews of prediction modelling studies (CHARMS) checklist [58, 59]. The recently published Prediction model Risk Of Bias ASsessment Tool (PROBAST) can be used to assess the risk of bias and applicability of CPRs [60]. Researchers can also assess the performance of existing CPRs on their own collected data [61]. Existing CPRs with potential should be updated, validated or tested in an impact study before a new CPR is developed [54, 62, 63]. If a new CPR is derived, researchers should clearly justify why it is required, with reference to existing CPRs, to avoid research waste and duplication of efforts [64]. Qualitative research with clinicians can be useful in determining whether a proposed CPR is clinically relevant, and to assess the credibility of the proposed predictor variables [65, 66].

### Stage 2: derivation of a clinical prediction rule according to methodological standards

Once a need for a new CPR is established, and a researcher has an appropriate clinical question, a CPR must be derived according to strict methodological standards [23]. There are various elements to consider, pertaining to the study design, statistical techniques employed and the assessment, presentation and reporting of the CPR. Researchers should consider writing and publishing a study protocol and registering the study prior to the derivation of a new CPR, in the interests of transparency [67, 68].

#### Study design for the derivation of a clinical prediction rule

The first stage in the development of a CPR is the derivation of the rule. This involves an examination of the ability of multiple potential variables from the clinical findings, history or investigation results to predict the target outcome of interest. Predicted probabilities are derived from the statistical analysis of patients with known outcomes, and the outcome of interest serves as the reference standard by which the performance of the CPR is assessed. The performance of a CPR is dependent upon the quality of the underlying data, and the dataset used to derive the CPR should be representative of the target population it is intended for [17, 30, 69, 70].

The optimal study design for the derivation of a diagnostic CPR is a cross-sectional cohort study, while for prognostic CPRs, the preferred design is a longitudinal cohort study [30]. In general, case-control studies are inappropriate, as they do not allow for the estimation of absolute outcome risk [21, 23, 71]; however, nested case-control or case-cohort studies can be used [71, 72]. Prospective cohort studies are preferred to retrospective cohort studies, to optimise measurement and documentation of predictive and outcome variables [21, 23]. For prescriptive CPRs, study designs that include a control group, such as randomised controlled trials (RCTs), are essential to ensure that treatment effect modifiers and non-specific prognostic predictors are distinguishable from one another [73, 74]. The study design should be adequately detailed and include the study setting, inclusion and exclusion criteria and patient demographics and characteristics [17]. To enhance generalisability, multicentre studies are recommended [30].

#### Statistical analysis

Commonly used statistical methods for the derivation of CPRs include multivariable regression techniques, and recursive partitioning techniques, such as classification and regression tree analysis [75]. Methods based on univariable analysis, where individual risk factors are simply totalled and assigned arbitrary weightings, should be avoided, as they are much less accurate than methods based on multivariable analysis [76]. This is because the final model may include predictors that are potentially related to each other and not independently associated with the outcome of interest [76]. Multivariable methods overcome the limitations of univariable analysis by enabling improved assessment of the association of the predictors with the target outcome [76].

In the case of multivariable regression, logistic regression models are required to predict binary events such as the presence or absence of a condition, while Cox regression models are suitable for time-to-event outcomes. Such models estimate regression coefficients (e.g. log odds or hazard ratios) of each predictor. Regression coefficients are mutually adjusted for the other predictors, and thus represent the contribution of each predictor to the probability of the outcome [23]. The probability of an outcome can be computed for a patient by combining the observed values of the predictors and their corresponding regression coefficients with the model intercept, or estimated baseline hazard [23]. For logistic models, the model intercept and the weighted values applicable to each patient are summed [16]. Specific values are assigned to each predictor, which are multiplied by the corresponding coefficients. In the case of a model with only binary categorical predictors, the predictors are multiplied by 0 or 1, depending on whether they are absent (0) or present (1), as per the model in Table 3 [77]. Exponentiating the final risk score gives the odds, and the probability (absolute risk) is calculated by use of the inverse logistic link function [78]. In this way, the probability of an outcome can be estimated from any combination of the predictor values [36]. The estimated probability for an individual without any of the predictors depends only on the intercept [23]. In this case, the value for each of the predictors will be 0; when each of these is multiplied by its relevant coefficient the value of 0 is retained [78]. For Cox regression models, the baseline hazard is estimated separately [26, 29].

**Table 3:** Clinical prediction rule for postoperative nausea and vomiting (PONV) [77]

Risk of PONV = 1/(1 + exp. − [2.28 + 1.27 × female sex + 0.65 × history of PONV or motion sickness + 0.72 × non-smoking + 0.78 × postoperative opioid use])	

Recursive partitioning involves repeatedly splitting patients into subpopulations including only individuals with a specific outcome [79], and was the method used to derive the Ottawa Ankle Rule [80]. CPRs can also be derived using discriminant function analysis [3], and machine learning algorithms based on artificial neural networks [1]. Artificial intelligence and machine learning approaches are becoming increasingly more common [81, 82].

#### Missing data

In clinical research, investigators almost always encounter missing observations involving predictor or outcome variables, even in carefully designed studies and in spite of their best efforts to maximise data quality [83]. There are three types of missing data mechanisms: (1) missing completely at random (MCAR), (2) missing at random (MAR) and (3) missing not at random (MNAR) [84]. When data are MCAR, this means that there are no systematic differences between the missing and observed values; for example, laboratory tests may be missing because of a dropped test tube or broken equipment. When data are MAR, this means that the probability of a missing value depends on the observed values of other variables (but not the unobserved values); for example, missing blood pressure measurements may be lower than observed measurements because younger people may be more likely to have missing measurements; in this case, data can be said to be MAR given age [85]. When data are MNAR, this means that the probability of a missing value depends on the unobserved values or other unobserved predictors, conditional on the observed data; for example, people with high blood pressure may be more likely to miss a doctor’s appointment due to headaches [85]. Missing values are rarely MCAR, that is, their ‘missingness’ is usually directly or indirectly related to other subject or disease characteristics, including the outcome [23, 25]. Missing data is frequently addressed with case-wise deletion, which excludes all participants with missing values from the analysis [85]. However, when data are plausibly MAR, this reduces sample size and statistical power and biases the results [85], leading to inaccurate estimates of predictor-outcome relationships and the predictive performance of the model, since the participants with complete data are not a random subsample of the original sample [84, 86, 87].

Multiple imputation is a popular approach to the problem of missing data [83, 85, 86, 88–91], as it quantifies the uncertainty in the imputed values, by generating multiple different plausible imputed datasets, and pooling the results obtained from each of them [85, 91]. Multiple imputation involves three stages [85, 89, 91–93]. First, as the name suggests, multiple imputed datasets are created, based on the distribution of the observed data. This first stage accounts for uncertainty in estimating the missing values by adding variability into the values across the imputed datasets. In the second stage, standard statistical techniques are used to fit the models that are of interest in the substantive analysis to each of the imputed datasets. Estimated associations in each of the imputed datasets will be different, due to the variability introduced in stage one. In the third and final stage, the multiple results are averaged together, and standard errors are calculated using Rubin’s combination rules [91], which account for both within-and between-imputation variability and the number of imputed datasets, and therefore the uncertainty of the imputed values. Multiple imputation typically assumes that data are MAR [93]. Importantly, the MAR assumption is just that; an assumption, rather than a property of the data [85]. The MCAR assumption can be tested, but it is not possible to differentiate between MAR and MNAR from the observed data [26, 85]. Most missing data are expected to be at least partly MNAR [85, 94, 95]. Sensitivity analyses can help to determine the effect of different assumptions about the missing data mechanism; work in this area is ongoing [96–100]. Other statistically principled approaches to dealing with missing data have been developed, based on random effects models [101, 102], Bayesian methods or maximum likelihood estimation [103] or, where data are longitudinal, joint models [104, 105]. Guidelines for reporting on the treatment of missing data in clinical and epidemiological research studies have been suggested by Sterne and colleagues [85]. Guidance also exists for handling missing data when deriving and validating CPRs [83, 106, 107]. It has been demonstrated that the outcome should be used for imputation of missing predictor values [87]. It is also becoming increasingly apparent that a real-time strategy to impute missing values is desirable when applying a CPR in clinical practice [108–110]. This is because one or more predictor variables may be unobserved for a particular patient, and thus the CPRs risk prediction cannot be estimated at the time of decision-making [108]. Real-time multiple imputation is not typically straightforward, as it requires access to the derivation dataset via, for example, a website [108, 110]. Of note, although multiple imputation is a widely advocated approach for handling missing data in CPR studies, a recent study showed that implementing simpler imputation methods resulted in similar predictive utility of a CPR to predict undiagnosed diabetes, when compared to multiple imputation [111].

#### Selection of candidate predictors for inclusion in a multivariable model

Candidate predictors are variables that are preselected for consideration in a multivariable model, and differ from those that are subsequently selected for inclusion in the final model [23]. Candidate predictors should be selected without studying the predictor-outcome relationship in the data; in other words, predictors should not be excluded as candidates solely because they are not statistically significant in univariable analysis [25, 26, 29, 112–114]. Predictor variables do not have to be causally related to the outcome of interest [21, 115]. Effects modelled in studies examining *causality* are expressed with relative risk estimates such as odds ratios, while risk *predictions* are presented as probabilities on an absolute scale between 0 and 1. Relative risk estimates are used in prediction research to calculate an absolute probability of an outcome for a patient, as described above, and can also be reported alongside risk predictions. All variables thought to be related to the target outcome can be selected as candidate predictors for inclusion in a multivariable model; however, when the number of outcome events in the dataset is small, there is a risk of overfitting the data when a large number of predictor variables are included. Thus the CPR will perform well on the derivation data, but poorly on new data [29, 69, 113, 116]. CPRs with a smaller number of predictors are also easier to use in practice. To overcome this problem, only the most clinically relevant candidate predictors should be chosen from the larger pool of potential predictor variables, without looking into the data [5, 117]. In addition, sample size recommendations for studies deriving CPRs are often based on the concept of events-per-variable (EVP), whereby the researcher controls the ratio of the number of outcome events to the number of coefficients estimated prior to any data-driven variable selection [31]. A rule-of-thumb of ten EPV has been suggested [29, 31, 114, 118]. Simulation studies examining the effect of this rule-of-thumb have yielded conflicting results [119–123]. One study found that when the EPV was less than ten, there were a range of circumstances in which coverage and bias were within acceptable levels [119]. Another found that 20 EPV or more are required when low-prevalence predictors are included in a model [123], while another suggested that problems may arise even when the EPV exceeds ten, as CPR performance may depend on many other factors [120]. Research in this area continues to evolve, as new guidance is clearly needed to support sample size considerations for the derivation of CPRs [121]. Recently, van Smeden and colleagues have suggested that sample size should be guided by three influential parameters: the number of predictors, total sample size and the events fraction [122].

Relevant predictors may be chosen based on a combination of clinical experience, expert opinion surveys, qualitative studies and formal systematic reviews and meta-analyses of the literature [26, 33, 36, 65, 124]. Strategies for reducing the number of candidate predictors include removing those that are highly correlated with others, and combining similar predictors [29]. Other considerations include selecting predictors that will be readily available for clinicians to observe or measure in the target setting, and selecting predictors that are relatively easy to measure and demonstrate high inter-rater reliability between clinicians [17, 21]. In terms of handling continuous predictors, researchers strongly advise against converting continuous variables into categorical variables, due to information loss and reduced predictive accuracy [125–128]. Similarly, it should not be assumed that continuous variables have a linear relationship [127]. Instead, methods that permit more flexibility in the functional form of the association between the predictors and outcome should be considered [127, 129]; two common approaches are fractional polynomials and restricted cubic splines [130, 131]. However, if sample size is limited, assuming a linear relationship between continuous variables may make a model less sensitive to extreme observations.

Penalised regression can be used to alleviate the problem of overfitting [116]. This approach involves placing a constraint on the values of the estimated regression coefficients in order to shrink them towards zero [116]. This has the effect of yielding less extreme risk predictions, and thus may improve the accuracy of predictions when the CPR is applied in new patients [113, 132]. The two most popular penalised methods are ridge regression [133] and lasso regression [134]. Unlike ridge regression, lasso regression also selects predictors as a consequence of its penalisation [116]. Ridge regression is usually preferred when a set of pre-specified predictors is available, while lasso regression may be preferred if a simpler model with fewer predictors is required [116, 132].

#### Selection of predictors during multivariable modelling

There is no consensus regarding how predictors should be selected while developing the final model [25]. Two common strategies include the ‘full model approach’ and the ‘predictor selection approach’ [23]. An alternative approach, known as ‘all possible subsets regression’, is less commonly used [28]. In the full model approach, all previously identified candidate predictors are included, and no further analysis is performed. Although this approach precludes selection bias and overfitting, it requires in-depth knowledge about the most relevant candidate predictors [26, 29]. In the predictor selection approach, predictors are chosen either by ‘backward elimination’ or ‘forward selection’, based on pre-defined criteria. Backward elimination begins with all predictors in the model and removes predictors, while forward selection begins with an empty model, and predictors are added successively. All possible subsets regression can build models with combinations of predictors not generated by the standard forward or backward procedures, because every conceivable combination of predictors is assessed to find the best fitting model [135]. With all methods, a series of statistical tests are performed to assess the ‘goodness of fit’ between the different models. Models can be compared by setting a pre-defined significance level and using the log likelihood ratio test, or using other model selection criterion such as the Akaike information criterion, or the Bayesian information criterion [23, 25]. Backward elimination is favoured, as it allows for the assessment of the effects of all predictors concurrently, and can take into account all correlations between predictors [136, 137]. Multiple testing in all possible subsets regression can easily lead to overfitting. However, with all methods, the choice of significance level impacts upon the number of final predictors; the use of smaller significance levels (e.g. *p* < 0.05) produces models with fewer predictors at the risk of excluding potentially important predictors, while the use of larger significance levels (e.g. *p* < 0.25) may result in the inclusion of less important predictors [25].

Predictor selection by so-called automated, data-dependent significance testing may generate overfitted, ‘optimistic’ models, particularly when the derivation dataset is small [23, 28, 128, 138, 139]. Thus, the Akaike information criterion is preferred, as it discourages overfitting by comparing models based on their fit to the data and penalising for the complexity of the model [25]. In addition, it may be acceptable to retain a non-significant predictor in a model, if there is substantial evidence of its predictive ability in the literature [26].

#### Definition and assessment of predictor and outcome variables

To ensure that the CPR can be accurately applied in practice, predictor and outcome variables should be clearly defined, and outcome variables should be clinically important [17]. Predictor variables must be reliable to enable their assessment in clinical practice; reliability refers to the reproducibility of the findings by the same clinician (intra-rater reliability) or between different clinicians (inter-rater reliability). Some researchers recommend that the reliability of predictor variables be explicitly evaluated, and that only those demonstrating good agreement beyond that expected by chance alone should be considered for inclusion [17]. A recent study found that measurement error of predictor variables is poorly reported, and that researchers seldom state explicitly when the predictors should be measured, and the CPR applied [140]. Another study demonstrated that predictor measurement heterogeneity across settings can have a detrimental impact on the performance of a CPR at external validation [141]. Ideally, the outcome variable should be assessed independently of the predictor variables to avoid circular reasoning or ‘incorporation bias’, when the results of the CPR or its predictor variables are used in the determination of the outcome [142]. However, it is acknowledged that this is not always possible, particularly for conditions that require a consensus diagnosis based on all available patient information [143]. It is well known that misclassification in the outcome variable may cause serious problems with prediction accuracy [144, 145].

#### Internal validation

Prediction models are known to perform better in the dataset from which they are derived, in comparison to applying them in new but plausibly related patients [146, 147]. ‘Plausibly related patients’ may be defined as those who are suspected of having the same condition or who are at risk of the same outcome examined in the derivation study [148]. This enhanced performance occurs simply because a model is designed to optimally fit the available data [23]. The performance of a model is most likely to be overestimated when the derivation dataset is small, and uses a large number of candidate predictors. Therefore, regardless of the approaches used in the derivation stage of development, internal validation is required to examine and correct the amount of overfitting or ‘optimism’ in the model, and thus the stability of the model [23].

Internal validation does not validate a model itself, but the process used to fit the model [26, 29]. Optimism is estimated using the original derivation dataset only. A number of methods are available for this purpose, including split-sampling, cross-validation and bootstrapping. Split-sampling is the simplest method, and is performed by dividing the derivation dataset into a ‘training’ sample and a ‘test’ sample prior to modelling. The CPR is then derived using the training sample, and its performance is assessed using the test sample [20]. However, the test sample usually comprises one-third of the original derivation dataset and is likely to be relatively small, resulting in imprecise performance estimates [149, 150]. This approach also squanders the test data that could have been used in the derivation of the CPR [23, 150]. In cross-validation, the CPR is derived using the whole derivation dataset, and the whole dataset is then reused to assess performance [20]. It is randomly split into equal samples: five or ten samples are commonly used. In the case of five samples, the model is refitted using four of the five samples and its performance tested using the fifth; this process is repeated five times until each of the five samples has been used as the test data, and an average of the estimated performance is taken. To improve stability, the overall procedure can be replicated several times, using different random subsamples [149]. The preferred internal validation method is bootstrapping, particularly when the derivation dataset is small or a large number of candidate predictors are assessed [23, 29]. The idea is to mimic random sampling from the target population by repeatedly drawing samples of the same size with replacement from the derivation dataset [151]. Sampling with replacement renders bootstrap samples similar, but not identical, to the original derivation sample [23]. Each step of model development is repeated in each bootstrap sample (typically 500), most likely yielding different models with varying performance. Each bootstrap model is then applied to the original derivation sample, yielding a difference in model performance. The average of these differences indicates the optimism in the performance metrics of the model that was initially derived in the derivation dataset [23, 26, 29, 151], and enabling adjustment of the overall performance to better approximate the expected model performance in novel samples [23]. Bootstrapping also estimates a uniform shrinkage factor to enable adjustment of the estimated regression coefficients for over-fitting [26, 29, 151]. However, no internal validation procedures can be a substitute for external validation; internal validation only addresses sampling variability, while external validation considers variation in the patient population [147].

#### Clinical prediction rule performance measures

CPR predictive performance can be assessed in terms of overall performance, calibration and discrimination [26]. ‘Overall performance’ can be quantified by calculating the distance between observed and predicted outcomes, using measures such as *R*^2^ or the Brier score [152]. ‘Calibration’ reflects the agreement between the predicted probabilities produced by the model and the observed outcome frequencies [23]. For example, if a model predicts a 20% probability of residual tumour for a testicular cancer patient, residual tumour should be observed in about 20 out of 100 of these patients [46]. ‘Internal calibration’ refers to agreement between predicted probabilities and observed outcome frequencies in the derivation dataset, where poor calibration may indicate lack of model fit or model misspecification [153]. ‘External calibration’ refers to agreement between predicted probabilities and observed outcome frequencies in novel datasets external to the one from which the model was derived, where poor calibration may indicate an overfitted model [153]. Calibration can be visualised by categorising individuals into quantiles based on their predicted probabilities, and plotting the observed outcome frequencies against the mean predicted probabilities [25]. Such a plot is the graphical equivalent of the Hosmer and Lemeshow goodness-of-fit test [154], which, although frequently used, may lack statistical power to identify overfitting [25, 26]. Alternatively, binary outcomes can be regressed on the predicted probabilities of the fitted model to estimate the observed outcome probabilities using smoothing techniques such as the loess algorithm [29, 153]. A comprehensive overview of calibration is given in Van Calster et al. [155].

Discrimination reflects the ability of a CPR to discriminate between patients with, and without, the outcome of interest. The predicted probabilities for patients *with* the outcome should be higher than the predicted probabilities for those who do not have the outcome [46]. The easiest way to assess discrimination is by calculation of the discrimination slope, which is simply the absolute difference in the average predicted probabilities for patients with and without the outcome [26]. Discrimination can also be visualised with a simple box plot. The most widely used measure to assess discrimination is the concordance index (c-index) [156], or, for logistic models its equivalent, the area under the receiver operating characteristic curve (AUROC) [157]. These measures represent the chance that, given one patient with the outcome and one without, the CPR will assign a higher predictive probability to the patient with the outcome compared to the one without. A c-index or AUROC of 0.5 indicates predictions that are no better than random predictions, and a value of 1 represents perfect discrimination between patients with and without the outcome [29]. In theory, a CPR may demonstrate good *discrimination* (classifying patients into the correct risk categories), but poor *calibration* (inaccurately estimating the absolute probability of an outcome), and vice versa [158]. A model that cannot discriminate between patients with and without the outcome has little use as a CPR; however, poor calibration can be corrected without compromising discriminatory performance [19, 114]. Van Calster and Vickers [159] found that poorly calibrated models diminish the clinical usefulness of a CPR, and can be harmful for clinical decision-making under certain circumstances, emphasising the importance of developing well-calibrated CPR’s. On the other hand, a CPR with poor calibration but good discrimination at a particular risk threshold may be appropriate if the aim is to prioritise patients for assessment or treatment, by identifying those with a very low risk of the target outcome relative to the rest of the population [160].

Performance measures such as sensitivity, specificity, positive and negative predictive values and positive and negative likelihood ratios are used to assess performance following the application of a risk threshold. Choosing a risk threshold can often be arbitrary, and it can therefore be useful to consider a range of thresholds when assessing performance [19]. Ideally, a CPR will have both a high sensitivity and a high specificity, and therefore correctly identify the majority of patients who truly have the condition, as well as correctly exclude the majority of patients who do not actually have the condition. However, this scenario rarely occurs in clinical practice. More often than not, the definition of a threshold is based on clinical considerations about the relative consequences of false positive and false negative classifications. Sensitivity and specificity are inversely proportional, so that as sensitivity increases, specificity decreases and vice versa [161]. Defining a high cut-off point will result in good specificity and few false positives, but poor sensitivity and many false negatives. A test with a high specificity is useful for ruling in a disease if a person tests positive. This is because it rarely misdiagnoses those who do not have the condition of interest. Defining a low cut-off point will result in good sensitivity and few false negatives, but poor specificity and many false positives. A test with a high sensitivity is useful for ruling out disease if a person tests negative. This is because it rarely misdiagnoses those who have the condition of interest [161]. Receiver operating characteristic (ROC) curves display the sensitivity and specificity of a CPR across the full range of cut-off values, and can be used to choose an optimal cut-off threshold [162]. Other approaches to determining clinical cut-offs have also been proposed [163].

In recent years, some novel model performance measures have been proposed that quantify the clinical usefulness of a CPR, by taking into account the costs and benefits of clinical decisions. These measures include relative utility curves and decision curves [164, 165]. Decision curves in particular are becoming a popular method of evaluating whether clinical decisions based on CPRs would do more good than harm [166]. Decision curve analysis assumes that a given probability threshold is directly related to the cost to benefit ratio, and uses this threshold to weight false positive and false negative predictions. The cost to benefit ratio thus defines the relative weight of false-positive decisions to true-positive decisions [164]. Model performance can subsequently be summarised as a net benefit, by subtracting the proportion of false-positive patients from the proportion of true-positive patients, weighting by the relative costs of a false-positive and a false-negative result. The net benefit of a CPR can be derived across and plotted against the whole range of threshold probabilities, yielding a decision curve, similar to ROC curves that plot the full range of cut-offs for a sensitivity/specificity pair [164].

#### Presentation of a clinical prediction rule

The final step in the derivation of a CPR is to consider the format in which it should be presented. It is imperative that the regression coefficients and intercept of a final model are presented, and confidence intervals around predicted probabilities can also be provided [23, 26]. If the final regression formula (as in Table 3) is not provided, a CPR could not be applied by future users [36]. A model can be developed into a simple web-based calculator or application to enhance the usability of a CPR. This may be beneficial for complex CPRs, and would facilitate their integration into the electronic health record, allowing them to be used at the point of clinical care [167]. Nomograms, graphical decision trees and other novel visualisation techniques could also be used [26, 168], which may aid in the interpretation and understanding of a CPR [168]; however, these must be presented alongside the full model formula. Scoring systems are often used to simplify CPRs and facilitate use, where regression coefficients are converted to integer point values that can be easily totalled and related back to the predicted probabilities [169]. However, this transformation leads to a loss of information and therefore reduced predictive accuracy [170].

#### Reporting the derivation of a clinical prediction rule

Numerous systematic reviews have shown that reporting of the derivation of CPRs is deficient [6–8]. As a result, the TRIPOD guidelines were produced [36], and should be followed by all researchers working in this field.

### Stage 3: external validation and refinement of a clinical prediction rule

As previously noted, CPRs perform better in the dataset from which they are derived compared to their application in plausibly related or ‘similar but different’ individuals, even after internal validation and adjustment [24]. Diminished performance can be due to overfitting, unsatisfactory model derivation, the absence of important predictors, differences in how the predictor variables are interpreted and measured, differences in the patient samples (‘case mix’) and differences in the prevalence of the disease [26, 148]. There is no guarantee that even well-developed CPRs will be generalisable to new individuals. In one external validation study, a CPR to detect serious bacterial infections in children with fever of unknown source demonstrated considerably worse predictive performance, such that it was rendered useless for clinical care [146]. It is therefore essential to assess the performance of a CPR in individuals outside the derivation dataset; this process is known as external validation [28].

External validation is not simply repeating the steps involved at the derivation stage in a new sample to examine whether the same predictors and regression coefficients are obtained; neither is it refitting the model in a new sample and comparing the performance to that observed in the derivation sample [24, 31]. External validation involves taking the original fully specified model, with its predictors and regression coefficients as estimated from the derivation study; measuring and documenting the predictor and outcome variables in a new patient sample; applying the original model to these data to predict the outcome of interest; and quantifying the predictive performance of the model by comparing the predictions with the observed outcomes [20]. Performance should be assessed using calibration, discrimination and measures to quantify clinical usefulness such as decision curve analysis [164]. A CPR can also be refined if it demonstrates poor performance in an external validation study. Regrettably, few CPRs are externally validated [27, 171, 172]. A systematic review of CPRs for children identified 101 CPRs addressing 36 conditions; of these, only 17% had narrow validation and only 8% had broad validation [171].

#### Study design for the external validation of a clinical prediction rule

Ideally, a validation study should be conducted prospectively, by enrolling new individuals in a specifically predesigned study, and the CPR should be applied to all patients meeting the study inclusion criteria [17, 23]. However, validation studies can be conducted retrospectively, using existing datasets. If adequate data on the predictor and outcome variables is available [23]. Investigators conducting a validation study should receive brief training on the accurate application of the CPR. If possible, all patients should be subjected to the reference standard, to establish their true outcome and enable comparison with the CPR prediction. However, in some cases, this may not be feasible or practical, and an appropriate and sensible proxy outcome may be used instead [173]. Stiell and Wells [17] recommend that the inter-rater reliability of the interpretation of the CPR result is assessed, to determine if the CPR is being applied accurately and consistently. In terms of sample size, for a logistic regression model with six predictors, a minimum of 100 patients with the outcome of interest and 100 patients without the outcome of interest has been suggested [174]. Other authors propose that external validation studies require a minimum of 100 events, but ideally 200 events [175]. A minimum of 200 events and 200 non-events has been suggested in order to reliably assess moderate calibration and produce useful calibration plots [155]. The characteristics of patients included in a validation study should be described in detail, and compared with those included in the derivation study. To enhance the interpretation of external validation studies, it is possible to quantify the degree of relatedness between derivation and validation datasets, to determine the extent to which the CPR can be generalised to different populations [34]. Authors have also proposed benchmark values to distinguish between a case-mix effect and incorrect regression coefficients in external validation studies, and therefore assist in the interpretation of a CPR’s performance in validation samples [176]. Similarly, a model-based concordance measure has recently been derived that enables quantification of the expected change in a CPR’s discriminative ability owing to case-mix heterogeneity [177].

#### Types of external validation

Many types of external validation are recognised in the literature, but all types consider patients that differ in some respect from the patients included in the derivation study [26]. The greater the differences between the patients in the derivation and validation samples, the stronger the test of generalisability of the CPR [24]. Three types of external validation have received the most attention, namely *temporal* validation, *geographical* validation and *domain* validation [148].

In *temporal* validation studies, the CPR is tested on patients in the same centre(s) but over a different time period [147]. *Geographical* validation studies examine the generalisability of the CPR to other centres, institutes, hospitals or countries [147]. Patient characteristics are likely to vary between locations, and predictor and outcome variables are likely to be interpreted and measured differently in different places, leading to greater differences between the derivation and validation populations than in a temporal validation study [24, 148]. In *domain* validation, the CPR is tested in very different patients than those from whom it was derived, for example in patients from a different setting (e.g. primary or secondary care), or in patients of different ages (e.g. adults vs. children). The case mix of patients included in a domain validation study will clearly differ from the derivation population [148]. Differences between the derivation and validation populations are generally smallest in a temporal validation study, and greatest in a domain validation study; therefore, good performance of a CPR in a temporal validation study may only provide weak evidence that the CPR can be generalised to new patients, while good performance in a domain validation study can be considered as the strongest evidence of generalisability [148]. Other types of external validation studies include *methodologic* validation which refers to testing using data collected via different methods, *spectrum* validation which refers to testing in patients with different disease severity or prevalence of the outcome of interest and fully *independent* validation which refers to testing by independent investigators at different sites [26, 147]. A recent study of cardiovascular risk CPRs found that very few were externally validated by independent researchers; to increase the chance of fully independent validation, researchers should report all the information required for risk calculation, to ensure replicability [178]. Some authors have found that CPRs demonstrate worse performance in fully independent external validation studies compared to temporal or geographical external validation studies [26, 28], while others have found no difference [179]. When multiple external validations of a CPR have been performed, it is useful to conduct a formal meta-analysis to summarise its overall performance across different settings and to assess the circumstances under which the CPR may need adjusting; a recently published framework provides guidance on how to do this [35].

#### Refinement of a clinical prediction rule: model updating or adjustment

When researchers encounter an inferior performance of a CPR in an external validation study compared with that found in the derivation study, there is a temptation to reject the CPR and derive an entirely new one in the often considerably smaller validation dataset [148, 180]. This approach leads to a loss of scientific information captured in the derivation study and an abundance of CPRs developed for the same clinical situation, leaving clinicians in a quandary over which one to use [24, 148]. However, a reduction in performance is to be expected in an external validation study [24, 26, 148]. The recommended alternative is to update, adjust or recalibrate the CPR using the validation data, thereby combining information captured in the original CPR with information from new patients and improving generalisability [22, 181, 182]. Several methods for updating CPRs are available. When the outcome prevalence in the validation study is different to that in the derivation study, calibration in the validation sample will be affected, but can be improved by adjusting the baseline risk (intercept) of the original model to the patients in the validation sample [180]. If the CPR is overfitted or underfitted, calibration can be improved by simultaneously adjusting all of the regression coefficients [24]. To improve discrimination, individual regression coefficients can be re-estimated, or additional predictors can be added [24, 180]. Ideally, updated CPRs that are adjusted to validation samples should themselves be externally validated, just like newly derived CPRs [148].

#### Comparing the performance of clinical prediction rules

Once a CPR has been externally validated, it is useful to compare its performance with the performance of other existing CPRs for the same condition [61]. Improvements in discrimination can be assessed by quantifying the difference in the AUROC or equivalent c-index between two CPRs [183]; however, this approach is inappropriate in the case of nested models that are fitted in the same data set [184]. Novel metrics have been proposed that quantify the extent to which a new CPR improves the classification of individuals with and without the outcome of interest into predefined risk groups [46]. These include the net reclassification improvement (NRI), and the integrated discrimination improvement (IDI) [185]. Various decision-analytic approaches to model comparison have also been proposed [186]. All of these measures can be used for comparing both nested and non-nested models. However, both the NRI and IDI statistics have come under intense scrutiny in the literature and many researchers caution against their use, as positive values may arise simply due to poorly fitted models [30, 187–191]. Therefore, the NRI and IDI statistics cannot be recommended [192]. Decision-analytic methods are increasingly recommended as they incorporate misclassification costs and therefore indicate the clinical usefulness of CPRs [186]. A systematic review of comparisons of prediction models for cardiovascular disease found that formal and consistent statistical testing of the differences between models was lacking and that appropriate risk reclassification measures were rarely reported [193]. A recent commentary provides a useful and comprehensive overview of the advantages and disadvantages of the various methods available for quantifying the added value of new biomarkers [194].

#### Reporting the external validation of a clinical prediction rule

External validation studies of CPRs are often poorly reported [9]; researchers should adhere to the TRIPOD checklist and accompanying guidelines [36].

### Stage 4: impact of a clinical prediction rule on clinical practice

Since the ultimate aim of a CPR is to improve the quality of patient care, the effect of a validated CPR on clinician behaviour and patient outcomes should be examined in what are known as impact analysis studies [22, 24]. It is increasingly recognised that CPR’s should be regarded as complex interventions, as the introduction of a CPR into clinical practice with subsequent management decisions consists of multiple interacting components [108, 195–201]. The impact of a CPR on clinical practice will depend on several interacting factors, including the accuracy and applicability of the CPR, clinicians’ interpretation of probabilities and clinicians’ adherence to and acceptance of the CPR [196]. Evaluating the impact of a CPR has been described as ‘the next painful step’ in the development process [202]. Impact analysis studies clearly differ from validation studies as they must be comparative, typically requiring a control group of clinicians providing usual care [22, 24, 32]. It is possible to assess the impact of both assistive CPRs that simply provide predicted probabilities, and directive *decision* rules that suggest a specific course of action based on probability categories [32]. Assistive CPRs respect clinicians’ individual judgement and leave room for intuition, whereas directive rules may be more likely to influence clinician behaviour [32, 203, 204]. However, it is not guaranteed that clinicians will follow CPR, or the recommendations provided by directive rules [32]. Therefore, an impact study must demonstrate that clinical behaviour can be altered and patient care improved by the CPR, prior to widespread dissemination and implementation [17].

Unfortunately, even fewer CPRs undergo an impact assessment than undergo external validation. In the systematic review of 101 CPRs for children, none had impact analysis performed [171]. An evaluation of 434 primary care CPRs found that only 12 had undergone impact analysis [172]. A subsequent systematic review of the impact of primary care CPRs found 18 studies relating to 14 CPRs, with 10/18 studies demonstrating an improvement in primary outcome when the CPR was used compared to usual care [205]. This review cautioned that the small number of impact analysis studies found precluded the possibility of drawing firm conclusions about the overall effectiveness of CPRs in primary care, with the authors pointing out that the methodological quality of the included studies was unclear due to incomplete reporting [205]. Another recent systematic review of the impact of CPRs found that the intermediate consequences of a CPR such as clinical management decisions were the primary outcome in the majority of studies, while few studies aimed to establish the effect of a CPR on patient outcomes [206]. In addition, in many of the included studies, the risk of bias was either high or unclear [206]. Finally, a study describing the distribution of derivation, validation and impact studies in four reviews of leading medical journals since 1981 demonstrated that a minority of studies concerned CPR impact (10/201), with the pattern remaining stable over time [27].

#### Study design for an impact analysis

Before carrying out a formal impact study, researchers must consider whether the CPR is ready for implementation [108, 207]. If possible, the predictive performance of the CPR should be verified in the new setting, and the CPR tailored to the new setting to enhance performance [108]. The optimal study design for an impact analysis is a cluster randomised trial with centres as clusters [22]. Randomising individual patients is not recommended as clinicians may learn the rule and apply it to patients randomised to the control group [22]. Randomising clinicians is preferable but requires more patients, and may lead to contamination of experience between clinicians in the same centre [24, 208]. An attractive variant of a cluster randomised trial is the stepped-wedge cluster randomised trial. In a stepped-wedge design, all centres apply care-as-usual, and then use the CPR at different, randomly allocated time periods [209]. This design allows for the comparison of outcomes both within and between hospitals, generates a wealth of data regarding potential barriers to implementation and is particularly beneficial if the CPR turns out to have a promising effect [210]. When the outcome of interest in an impact study is clinician behaviour or decision-making, a cross-sectional randomised study without patient follow-up is sufficient, with randomisation at either the patient or clinician level. However, to determine the impact of a CPR on patient outcomes or cost-effectiveness, follow-up of patients is essential [22].

Given the significant practical, logistic and economic challenges associated with cluster randomised trials, non-randomised approaches are possible and are often used. Cluster randomised trials can be expensive and time-consuming and it may be difficult to recruit an adequate number of clusters [24, 108]. A suggested rule-of-thumb is to regard four clusters per arm as the absolute minimum number required [211]; however, methods for determining sample size in cluster randomised trials have been proposed by a number of authors [212–214]. A popular design is a before–after study, in which outcomes are assessed in a time period before a CPR is available and compared with outcomes measured in a time period after it is introduced; this design is susceptible to temporal confounding [24]. Finally, a relatively low-cost and simple design is a before–after study within the same clinicians. In this design, clinicians are asked to indicate their treatment or management decision or perceived risk of disease for the same patient both before, and after, receiving the CPR prediction [24]. Single centre impact studies are recommended to inform the planning of multicentre randomised trials [32]. As with derivation and validation studies, a sample size calculation should be performed, with consideration of all relevant impact measures, and where possible assessment of outcome measures should be blinded to the CPR predictions and recommendations [32, 33]. Clinicians must undergo training in order to correctly interpret and use the CPR [17].

The impact of CPRs can also be estimated indirectly using decision analytic modelling, which integrates information on CPR predictions and information about the effectiveness of treatments from therapeutic intervention studies [215, 216]. Such studies cost less, and take less time, than RCTs; however, they are limited by the quality of available evidence, and only provide theoretical indications of the impact CPRs may have on patient outcomes. Thus it has been suggested that they should not replace RCTs but rather be performed as an intermediate step prior to an RCT [217].

#### Measures of impact of a clinical prediction rule

During an impact analysis study, the sensitivity and specificity of the CPR should be recalculated to determine its accuracy in the new study population [17]. However, measures of CPR *accuracy* are not synonymous with measures of *impact*, and only represent the *potential* impact of the CPR [32]. This is because clinicians are unlikely to follow the logic of the CPR or its recommendations in every case; they may not use the CPR at all, they may not use it correctly, they may deliberately disregard its predictions or suggestions or they may be unable to use it for other reasons [32]. Measures that are assessed in traditional RCTs include safety, which refers to any adverse events resulting from the implementation of an intervention, and efficacy, which relates to the extent that an intervention helps to improve patient outcomes, for example by reducing mortality rates [218]. In addition, Reilly and Evans [32] propose that the impact of a CPR is assessed in terms of its ‘safety’ and ‘efficiency’, where safety is defined as the proportion of patients found to have the outcome of interest and who received the appropriate intervention, and efficiency is defined as the proportion of patients *without* the outcome of interest and who *did not* receive the intervention. The sensitivity and specificity of a CPR will only be the same as its safety and efficiency if clinicians follow the logic and recommendations of the CPR exactly [32]. Therefore, in an impact analysis study, a CPR may demonstrate more, or less, actual impact than its potential impact. The effect of clinicians’ incorrect use of the CPR, or their deviations from its logic or suggestions can provide important insights into its impact under specific circumstances, and may reveal complex interactions between clinicians and the CPR [32]. For example, Reilly and colleagues [219] found that when clinicians did not consult a CPR for suspected acute cardiac ischemia at all, or overruled its recommendations, their decisions were less efficient than if they had followed the CPR in every case.

#### Acceptability of a clinical prediction rule

If the use of a CPR is warranted but it is not used, the considerable time, money and effort that goes into its development and evaluation is wasted. Assessing the acceptability of a CPR is therefore crucial for successful implementation. Even valid and reliable CPRs may not be accepted or used by clinicians [17]. Impact studies allow researchers to evaluate the acceptability of a CPR to clinicians, patients or others who may use it, as well as its ease of use and barriers to its uptake [22]. If a CPR proves to be acceptable, its long-term and widespread dissemination and implementation would be justified; if not, the CPR could undergo modification and further evaluation [48]. Acceptability of a CPR and attitudes towards it can be determined via survey, qualitative, simulation or clinical vignette studies [33, 48, 220–222]. The validated Ottawa Acceptability of Decision Rules survey instrument can be used both to measure the overall acceptability of a CPR, and to assess specific barriers to its use, which can inform potential improvements to the CPR as well as the design of dedicated implementation strategies [48]. Qualitative studies can be invaluable for determining the acceptability of a CPR but are relatively rare [200, 220, 222–225].

#### Comparison of a clinical prediction rule with unstructured clinical judgement

For a CPR to improve the diagnostic accuracy of clinicians, its performance in distinguishing between patients with and without the condition of interest should be superior to that of unstructured clinical judgement alone. Therefore, a vital metric is the comparison of the accuracy of the CPR-predicted probabilities of disease, or recommended decisions, with the accuracy of clinicians own estimated disease probabilities or management decisions [18]. The sensitivity and specificity of clinicians’ predictions or decisions are generally measured under usual practice, and compared to the sensitivity and specificity of the CPR predictions or decisions when applied to the same patients [226, 227]. Some studies have used clinical vignettes [228] while others have used multivariable logistic models to assess the added value of a CPR over and above clinical judgement alone [229]. If it can be demonstrated that the performance of a CPR is superior to unaided clinician judgement, this may aid clinicians’ acceptance and use of the CPR [32]. Although comparison of a CPR to clinician suspicion regularly takes place at the impact analysis stage, some researchers have recommended that this is carried out during the derivation or validation stages, arguing that if the CPR does not add anything beyond clinical judgement, then the use of the CPR and an impact study would not be warranted [230]. In addition, Finnerty and colleagues [231] recommend that comparison is undertaken in multiple settings, as the performance of a CPR may be superior to clinical judgement in certain settings, but inferior or no different in other settings. A recent systematic review comparing CPRs with clinical judgement concluded that the differences between the two methods of judgement are likely due to different diagnostic thresholds, and that the preferred judgement method in a given situation would therefore depend on the relative benefits and harms resulting from true positive and false positive diagnoses [232]. Brown and colleagues’ [200] found that the use and potential advantages of a CPR may be much more complex than originally thought, and that CPRs may be useful for purposes not previously reported, such as enhancing communication with colleagues and patients, and medico-legal purposes. Recent studies in the child protection field have demonstrated that CPRs may provide clinicians with additional confidence in their decision-making, even if they do not alter their management actions based on the CPRs risk prediction [220, 233].

#### The four phases of impact analysis for clinical prediction rules

**Fig. 2:**
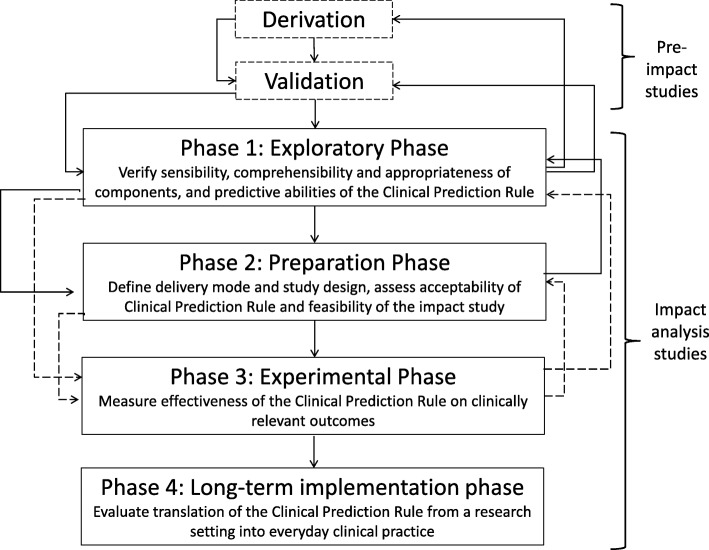
The four phases of impact analysis for a clinical prediction rule. Reproduced with permission from Wallace et al. 2011 [33]

Despite the abundance of methodological guidelines for the derivation and validation of CPRs [26], there is a lack of clear guidance for the design, conduct and reporting of impact analysis studies of CPRs. To this end, Wallace and colleagues [33] formulated an iterative four-phased framework for the impact analysis of CPRs, specifying the importance of substantial preparatory and feasibility work prior to the conduct of a full-scale formal experimental study (Fig. 2). Phase 1 involves determining whether the CPR is ready for impact analysis, i.e. whether it has been rigorously derived and broadly validated according to pre-defined methodological standards. Phase 2 includes assessing the acceptability of the CPR and identifying potential barriers to its uptake and implementation, as well as assessing the feasibility of conducting an impact study. Evaluating the feasibility of carrying out an impact study involves consideration of multiple factors including the most appropriate study design for measuring relevant outcomes, and how the CPR will be delivered at the point of care or integrated into the clinical workflow. Phase 3 involves formally testing the impact of the CPR using a comparative study design. Phase 4 involves long-term dissemination and implementation of the CPR, which corresponds to stage 6 in the development of CPRs, discussed below.

#### Reporting the impact analysis of a clinical prediction rule

There are currently no published reporting guidelines for studies analysing the impact of CPRs. This is a gap in the literature, and a priority for future research. However, researchers assessing the impact of CPRs in an RCT may refer to guidelines on the reporting of clinical trials, such as the Consolidated Standards of Reporting Trials (CONSORT) statement [218].

### Stage 5: cost-effectiveness of the clinical prediction rule

If an impact analysis study shows that a CPR demonstrates safety and efficiency, alters clinician behaviour and improves clinical care, a formal economic evaluation can be carried out to determine the cost-effectiveness of the CPR. The aim is to establish the health care savings associated with routine use of the CPR in clinical practice [17]. Economic evaluation is usually based on decision analytic models [234]. Any economic evaluation must make reasonable assumptions about the accuracy and effectiveness of the CPR and the costs involved [17]. Sensitivity analyses should be performed by re-running models with alternative assumptions, to examine the uncertainty of the model projections [234]. In reality, many economic evaluations are conducted prior to an impact analysis study or even an external validation study, perhaps because they are relatively quick and low cost to perform, and provide a significant part of the justification for the development and implementation of a CPR.

### Stage 6: long-term implementation and dissemination of the clinical prediction rule

The gap between evidence and practice has been consistently demonstrated in health services research [235], and there is no guarantee that a CPR will be widely disseminated or used, even if it is shown to have a positive impact on clinical care and cost benefits. Therefore, in order to maximise the uptake of a CPR, an active dissemination and implementation plan must be in place. Simple passive diffusion of study results via publication in journals or presentations at conferences is unlikely to significantly change clinical practice [236]. Examples of dissemination include actively targeting specific audiences via direct mail or the press, while implementation involves the use of local administrative, educational, organisational and behavioural strategies to put the CPR into effect in clinical practice [236]. Active broad dissemination of the widely accepted Ottawa ankle rule via an educational intervention found no impact of the rule on clinicians’ use of ankle radiography [237], leading the authors to recommend implementation strategies at the local level instead. Some implementation strategies have been found to be more effective than others in changing clinician behaviour. A systematic review found the most effective approaches to be reminders in the form of posters, pocket cards, sheets or computer-embedded prompts, face-to-face local clinician education and the use of multiple interventions simultaneously [238]. Incorporation of CPRs into clinical guidelines may also be of benefit; a recent study found that clinical guidelines and local policies that mandated the use of CPRs were effective in increasing their adoption in clinical practice [200]. In addition, the integration of CPRs into the clinical workflow via electronic health records may promote their use [239]. Since impact in a research study does not ensure impact in real-world clinical practice, follow-up of clinicians can be conducted to assess the long-term use and effect of the CPR [17, 33].

#### Barriers and facilitators to the use of clinical prediction rules

Clearly, identifying the barriers and facilitators to the implementation of CPRs is crucial for the development of targeted implementation strategies that may encourage clinicians to use the CPR. The adoption of CPRs into clinical practice is influenced by various factors including clinician characteristics, patient factors, features of the CPR itself and environmental factors [32, 66, 221, 224, 225, 240–252].

Table 4 provides an overview of the barriers to the adoption of CPRs identified in the literature [253], grouped according to their effect on clinician knowledge, attitudes or behaviours [254]. Barriers relating to knowledge include lack of awareness of the CPR or the burden of the clinical problem it applies to, unfamiliarity with the CPR and a lack of understanding of the purpose of CPRs in general [225, 240–242]. Clinicians may also be unaware of a CPR due to the increasing volume of CPRs, particularly when they are developed for the same condition [61, 243]. Common barriers relating to clinician attitude include a conviction that clinical judgement is superior to the CPR, and distrust of the accuracy of the CPR [32, 224, 240, 241, 244, 245]. Barriers relating to behaviour include organisational factors [251], the complexity of the CPR and the time it takes to apply; survey studies suggest that clinicians much prefer a CPR that is simple to use, memorable and saves time [221, 246, 247]. Complex models such as those based on machine and artificial learning algorithms may introduce additional barriers relating to applicability and usability, due to their potential lack of reproducibility and transparency [60, 82]. Other studies have demonstrated that clinicians will be unlikely to use a CPR if there are predictors missing which are deemed to be important, or if the predictor variables are not logically related to the outcome variable [32, 225]. Reilly and Evans [32] offer a number of strategies for overcoming barriers to the use of CPRs. These include emphasising the discretionary use of the CPR, comparing clinical judgement with the CPR, checking whether any excluded factors affect the CPR predictions, performing a simulated impact analysis and soliciting clinicians input regarding the logic and format of the CPR, among others [32].

**Table 4:** Barriers to the use of clinical prediction rules in practice identified in the literature

Theme	Subtheme	Barrier
Knowledge	Awareness	Unaware:
		• That CPR exists
		• Of clinical problem or burden of clinical problem to which CPR applies
		Unable to choose from multiple CPRs
	Familiarity	Unfamiliar with CPR
	Understanding	Lack of knowledge and understanding of the purpose, development and application of CPRs in general
	Forgetting	Clinician forgets to use CPR despite best intentions
Attitudes	Negative beliefs about CPRs	Belief that:
		• CPRs threaten autonomy
		• CPRs are too ‘cook-book’, and oversimplify the clinical assessment process
		• Clinical judgement is superior to CPRs
		• Clinical judgement is not error prone
		• Use of CPRs causes intellectual laziness
		• The development of the CPR was biased
		• Patients will deem clinicians less capable if using a CPR
		• CPRs only apply to the less experienced
		• Probabilities are not helpful for decision-making
		Dislike of the term ‘rule’
		Clinician had a false negative result when using a CPR in the past
		Existing CPRs are not ready for clinical application
	Outcome expectancy	Belief that:
		• CPRs will not lead to improved patient or process outcomes
		• The information provided by the CPR is not sufficient to alter clinical decisions
		Clinician:
		• Fears unintended consequences of use
		• Is uncertain about using the CPR in patients with an atypical presentation
		• Worries that improving efficiency threatens patient safety
	Self-efficacy	Belief that the CPR is too difficult to use
		Clinician uncertain how to interpret or use CPR output
	Motivation	Clinician lacks motivation to use the CPR
Behaviour	Patient factors	Patients expectations are not consistent with the CPR
	Features of the CPR	Clinician:
		• Finds CPR too complicated
		• Finds CPR ‘too much trouble’ to apply
		Perception that:
		• The CPR is not an efficient use of time • The CPR does not have face validity or that important predictors are missing
		• The CPR does not fit in with usual work flow or approach to decision-making
		• The CPR is not generalisable to the clinician’s patient
		• The CPR is static and does not consider the dynamic nature of clinical practice
		• Overruling the CPR is often justified
		Data required for the CPR is difficult to obtain
	Environmental factors	Lack of:
		• Time
		• Organisational support
		• Peer support for use
		Perceived increased risk of litigation
		Insufficient incentives or reimbursement for use of the CPR

Adapted from Sanders 2015 [253]. *CPR* clinical prediction rule

## Summary

For CPRs to be useful in clinical practice, they must be properly planned [67], derived using appropriate statistical techniques [23] and externally validated in multiple settings and by independent investigators to determine their predictive accuracy [148]. In addition, CPRs must undergo impact analysis to determine their effect on clinician behaviour and relevant patient outcomes [22]. There are numerous factors to consider when deriving, validating and assessing the impact of a CPR including the study design, preparatory work, statistical analysis, modelling strategy, performance/impact measures, the presentation of the CPR and the reporting of the study methodology. New CPRs should only be derived when there is a clear clinical need for them [17]. There is an urgent need to change the focus from the derivation of CPRs, to the validation and impact analysis of existing ones [33]. The CPR must be presented in full, and the study methods reported adequately, to ensure its quality, risk of bias and clinical utility can be evaluated; the TRIPOD guidelines should be followed to ensure completeness of reporting requirements [36]. Feasibility and preparatory work is essential to determine whether a formal impact study of the CPR is warranted [33, 108], and survey and qualitative work should be undertaken to verify whether the CPR is acceptable and relevant to clinicians [48, 65, 220, 222]. If a CPR is found to have a positive impact on patient outcomes, its cost-effectiveness should be evaluated, and a targeted implementation and dissemination strategy devised, with consideration of possible barriers to implementation, to maximise uptake [17].

In summary, the development and evaluation of a robust, clinically useful CPR with high predictive accuracy is challenging, and research in the field concerning derivation, validation and impact evaluation continues to evolve. However, adhering to the existing methodological standards and recommendations in the literature at every step will help to ensure a rigorous CPR that has the potential to contribute usefully to clinical practice and decision-making.

## Abbreviations

AUROC: Area under the receiver operating characteristic curve
CPR: Clinical prediction rule
EPV: Events per variable
IDI: Integrated discrimination improvement
MAR: Missing at random
MCAR: Missing completely at random
MNAR: Missing not at random
NRI: Net reclassification improvement
RCT: Randomised controlled trial
ROC: Receiver operating characteristic curve
TRIPOD: Transparent Reporting of a multivariable prediction model for Individual Prognosis or Diagnosis
